# Extensive Thiol Profiling for Assessment of Intracellular Redox Status in Cultured Cells by HPLC-MS/MS

**DOI:** 10.3390/antiox11010024

**Published:** 2021-12-23

**Authors:** Jiandong Wu, Anna Chernatynskaya, Annalise Pfaff, Huari Kou, Nan Cen, Nuran Ercal, Honglan Shi

**Affiliations:** 1 Department of Chemical and Biochemical Engineering, Missouri University of Science and Technology, Rolla, MO 65409, USA; jiandong.wu@mst.edu (J.W.); chernatynskayaa@mst.edu (A.C.); huari.kou@mst.edu (H.K.); 2 Department of Chemistry, Missouri University of Science and Technology, Rolla, MO 65409, USA; arpvdc@mst.edu; 3 Department of Computer Science, Missouri University of Science and Technology, Rolla, MO 65409, USA; nancen@mst.edu

**Keywords:** thiol, glutathione, biomarker, HPLC-MS/MS, lens epithelial cells, cancer cells

## Abstract

Oxidative stress may contribute to the pathology of many diseases, and endogenous thiols, especially glutathione (GSH) and its metabolites, play essential roles in the maintenance of normal redox status. Understanding how these metabolites change in response to oxidative insult can provide key insights into potential methods of prevention and treatment. Most existing methodologies focus only on the GSH/GSH disulfide (GSSG) redox couple, but GSH regulation is highly complex and depends on several pathways with multiple redox-active sulfur-containing species. In order to more fully characterize thiol redox status in response to oxidative insult, a high-performance liquid chromatography with tandem mass spectrometry (HPLC-MS/MS) method was developed to simultaneously determine seven sulfur-containing metabolites, generating a panel that systematically examines several pathways involved in thiol metabolism and oxidative stress responses. The sensitivity (LOQ as low as 0.01 ng/mL), accuracy (88–126% spike recovery), and precision (≤12% RSD) were comparable or superior to those of existing methods. Additionally, the method was used to compare the baseline thiol profiles and oxidative stress responses of cell lines derived from different tissues. The results revealed a previously unreported response to oxidative stress in lens epithelial (B3) cells, which may be exploited as a new therapeutic target for oxidative-stress-related ocular diseases. Further application of this method may uncover new pathways involved in oxidative-stress-related diseases and endogenous defense mechanisms.

## 1. Introduction

Oxidative stress is caused by an imbalance between levels of antioxidants and reactive oxygen species and nitrogen species (ROS/RNS) in living systems. The resulting accumulation of ROS/RNS damages cellular components and is strongly implicated in many human diseases [1,2]. Glutathione (GSH) plays a major role in endogenous antioxidant defense through multiple pathways. For example, peroxides and hydroperoxides are reduced to the corresponding alcohols by glutathione peroxidase, forming glutathione disulfide (GSSG) [3]. 

**Figure 1:**
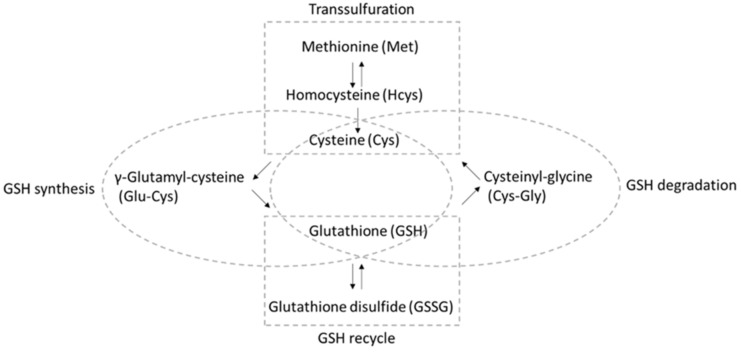
Analytes included in the “thiol profile” HPLC-MS/MS method described here and the pathways in which they participate.

A “dynamic balance” [4] involving several different pathways ensures that sufficient GSH is available for reaction with ROS/RNS (Figure 1) [5,6,7]. Aside from direct uptake and export [8], GSH can be recycled from GSSG by GSH reductase, with reducing equivalents from nicotinamide adenine dinucleotide phosphate (NADPH). Moreover, GSH can be synthesized de novo starting from cysteine (Cys), where the enzyme γ-glutamate cysteine ligase catalyzes the reaction between cysteine and glutamate to form γ-glutamyl-cysteine (Glu-Cys). Then, glycine is added to the C-terminus by GSH synthetase, forming the tripeptide γ-glutamyl-cysteinyl-glycine. It is worth noting that the sulfur-containing amino acid methionine (Met), along with the conversion of methionine to cysteine occurring via the transsulfuration pathway using homocysteine (Hcys) as the intermediate, is a significant source of cysteine [9]. Moreover, GSH can be degraded to cysteinyl-glycine (Cys-Gly), which is transported for the subsequent reutilization of Cys. 

Although the GSH/GSSG redox couple is the primary contributor to the cellular redox environment [10], there is increasing evidence that other thiols and methionine play important roles in the maintenance of cellular redox status, with dysregulation resulting in a variety of diseases [2,11,12,13,14,15]. The ability to quickly and conveniently profile thiol redox pathways in response to oxidative insult could further elucidate pathological mechanisms and, consequently, uncover potential therapeutic targets [16,17,18,19]. However, previous methods fall short in one or more important areas: compared to HPLC-UV or -FLD, LC-MS methods are capable of simultaneously measuring thiols (or their derivatives) and their corresponding disulfides with less sample workup, but most focus exclusively on the determination of GSH and GSSG [20,21,22,23,24]. One notable exception is a robust LC-MS/MS method developed by Sutton et al. for analyzing the thiol redox metabolome in blood, urine, and saliva [6]. However, spike recovery results were not reported; thus, the method was not fully validated, and its accuracy and precision in real samples are therefore questionable. 

As a result, researchers are not yet able to fully leverage the wealth of information contained in the “thiolome”. To address this knowledge gap, our group recently developed a HPLC-MS/MS method for the determination of several sulfur-containing analytes in tear fluid as a proximal indicator of redox status in deeper ocular tissues [7]. However, free thiols that were stable in tear fluids and/or diluted with mobile phase were found to autoxidize rapidly in cell homogenates, resulting in artifactually increased GSSG. To prevent this, the present method incorporates a derivatization step, wherein N-ethylmaleimide reacts with free thiols via Michael addition, forming stable adducts that can then be separated and quantified.

Therefore, we developed and validated a high-performance liquid chromatography with tandem mass spectrometry (HPLC-MS/MS) method for the simultaneous determination of six thiols and Met. This method provides a comprehensive “thiol profile” by probing the key metabolic pathways involved in the oxidative stress response and thiol recycling. The method was then used to obtain thiol profiles for four different cell lines, which demonstrated its utility in investigations of oxidative stress in a variety of tissues. In addition, this application revealed that different cell lines exhibit characteristic baseline thiol profiles, as well as distinctive responses to oxidative insult. 

## 2. Materials and Methods

### 2.1. Materials

L-Homocysteine was purchased from Advanced ChemBlocks Inc. (Burlingame, CA, USA); γ-glutamyl-cysteine was purchased from United States Biological (Salem, MA, USA); L-methionine was purchased from Bioworld (Dublin, OH, USA); L-cysteine was purchased from Ambeed (Arlington Heights, IL, USA); GSH, GSSG, and cysteinyl-glycine were purchased from Sigma Aldrich (St. Louis, MO, USA). *tert*-Butyl hydroperoxide solution (*t*BHP) was purchased from MilliporeSigma (St. Louis, MO, USA). Bradford reagent was purchased from BioRad (Hercules, CA, USA). N-Ethylmaleimide (NEM) was purchased from Chem-Impex International (Wood Dale, IL, USA). Molecular weight cut-off (MWCO) membrane centrifugal filter units (3 kD) were purchased from VWR (Radnor, PA, USA). Ultrapure water (18.2 MΩ∙cm) was prepared in-house using a Millipore Elix-3 purification system (Millipore, Billerica, MA, USA). For cell culture experiments, supplements and media were obtained from Thermo Fisher Scientific (Waltham, MA, USA), unless otherwise stated.

### 2.2. Standard Preparation

Methionine and GSSG stock solutions were prepared in ultrapure water while all others were prepared in aqueous NEM. The completion of thiol derivatization was confirmed by targeted analysis using HPLC-MS/MS [7]. Stock solutions were diluted in ultrapure water to prepare secondary standard solutions, which were kept at −20 °C. Calibration standards were prepared by serially diluting secondary stock solutions in mobile phase A (0.01% formic acid in water, *v*/*v*).

### 2.3. Cell Culture

Human lens epithelial cells (B3, ATCC CRL-11421) were purchased from ATCC (American Type Culture Collection, Manassas, VA, USA). Cells were grown in ATCC-formulated Eagle’s Minimum Essential Medium (# 30-2003, ATCC) supplemented with 10% heat-inactivated fetal bovine serum (FBS) and 1% penicillin/streptomycin/amphotericin B by following the protocol of the cell supplier. All experiments with B3 cells were performed in phenol red-free media, using tissue culture dishes coated with 20 μg/mL collagen type IV from human placenta (Advanced BioMatrix, San Diego, CA, USA).

A549 human lung carcinoma cells were kindly provided by Dr. Yue-Wern Huang from the Biological Sciences Department at Missouri University of Science and Technology. Cells were grown in phenol-red-free DMEM/F12 medium supplemented with 10% heat-inactivated FBS and 1% penicillin/streptomycin/amphotericin B. Serum- and growth-factor-free medium was used for all treatment experiments, instead of the fully supplemented media described above.

Human breast epithelial cells (MCF10A) were provided by the Cell Resources Core of the Barbara Ann Karmanos Cancer Institute (Detroit, MI, USA) and grown in DMEM/F12 medium supplemented with 5% horse serum and other supplements according to the protocol provided by the supplier. 

HN12 is a head and neck squamous cell carcinoma line derived from a lymph node metastasis. HN12 cells were kindly provided by Dr. Hu Yang from the Chemical and Biochemical Engineering Department at Missouri University of Science and Technology. Cells were grown in DMEM (Corning Inc., Corning, NY, USA) with 10% FBS (HyClone Lab, Logan, UT, USA) and 1% penicillin/streptomycin.

All the cells were grown in a humidified cell culture incubator maintained at 37 °C and 5% carbon dioxide.

### 2.4. Cell Sample Preparation

Cells were released by trypsin and centrifuged to form pellets. Cell pellets were washed with PBS twice before homogenization. For the experiments with *t*BHP treatment, 10 mM NEM in PBS, instead of PBS, was used to wash the pellets of all groups.

For homogenization, approximately 100 mg of zirconium oxide beads (0.5 mm diameter, Next Advance, Troy, NY, USA) and 10 mM NEM aqueous solution (approximately 200 µL NEM per 1 × 10^6^ cells) were added to the cell pellets. The cells were homogenized using a Bullet Blender Storm tissue homogenizer (Next Advance, Troy, NY, USA) at speed “10” for 5 min. Upon release from their intracellular compartments, free thiols were derivatized within seconds [6]. The resulting homogenates were immediately centrifuged at 5000× *g* for 10 min at 4 °C. For each sample, a 50 µL aliquot of supernatant was reserved for the determination of protein content via the Bradford method [25]. Another 25 µL of supernatant was transferred to an ultrafiltration unit pre-loaded with 475 µL mobile phase A and then centrifuged at 13,000× *g* for 30 min at 4 °C. The filtrate was injected for LC-MS analysis of all analytes except for GSH, which required an additional 5-fold dilution.

Spike recovery tests were performed at two levels for each analyte using B3 cell homogenates, prepared in triplicate. The spike standard (50 µL) along with the supernatant of cell homogenate (25 µL) was added to an ultrafiltration unit pre-loaded with 425 µL mobile phase A and then centrifuged at 13,000× *g* for 30 min at 4 °C. The recovery was calculated as (measured concentration of spiked sample—measured concentration of non-spiked sample)*/*spiked concentration.

### 2.5. Treatment with tBHP

*t*BHP was used to induce oxidative stress in vitro. *t*BHP solutions were freshly prepared from concentrated stock solutions immediately prior to each experiment [26]. For all experiments, the cells were seeded at 1 × 10^6^ cells per well on a six-well plate incubated in complete medium overnight prior to the experimental treatment. After overnight incubation, the medium was removed and replaced with the corresponding treatment media (plain media for control groups or *t*BHP diluted in plain media for treatment groups). After treatment, cells were then processed as described above.

### 2.6. HPLC-MS/MS Method

A Shimadzu (Columbia, MD, USA) Prominence UFLC system coupled to a 4000 Q TRAP tandem mass spectrometer system (AB SCIEX, Concord, ON, Canada) was used as previously described [27]. Analytes were separated via HPLC using a HydroRP column (4 μm, 250 × 2 mm) purchased from Phenomenex (Torrance, CA, USA) with mobile phase delivered at a binary flow rate of 0.3 mL/min using the following gradient program: 100% mobile phase A (0.01% formic acid in ultrapure water) for 0.5 min, followed by a linear gradient of 0–60% B from 0.5 to 6.0 min, then holding at 60% B from 6.0 to 7.0 min, and, finally, back to 100% A from 7.0 to 7.5 min, followed by a 4.5 min re-equilibration with 100% A before the next injection. The column oven temperature was set at 40 °C, and the injection volume was 20 μL.

Eluted analytes were detected and quantified using positive electrospray ionization in MRM mode. All mass spectrometer conditions were optimized for quantification of the analytes, and the following parameters were set accordingly. The ion source temperature was set to 600 °C, and the ion spray voltage was 5500 V. The curtain gas pressure was 15 psi, and the ion source gases (GS1 and GS2) were set to 20 and 45 psi, respectively.

### 2.7. Statistical Analysis

Intracellular analyte concentrations were normalized to the protein concentration in each sample and reported as mean ± SD for biological replicates. For the *t*BHP exposure experiments, differences between treatment groups (*t*BHP group vs. control group) were analyzed by Student *t*-test, with *p* < 0.05 considered statistically significant. Principal component analysis was performed using values after log transformation.

## 3. Results and Discussion

### 3.1. HPLC-MS/MS Method Performance and Validation

MS/MS parameters were optimized during direct infusion of each analyte, introduced at a rate of 0.6 mL/h via syringe pump. The resulting declustering potential (DP), collision energy (CE), and collision cell exit potential (CXP) for each analyte are shown in Table 1. Aside from the quantification ion pairs listed in Table 1, confirmation ion pairs (not shown) were also used for each analyte in the preliminary experiment to ensure that all analytes were correctly monitored.

**Figure 2:**
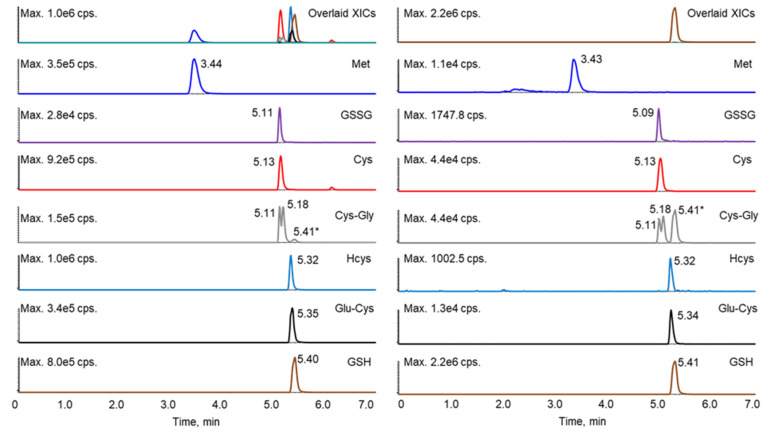
Extracted ion chromatograms of the standard mixture prepared at 200 ng/mL (left) and a representative sample (right). * The peak at 5.41 min in the Cys-Gly panel (grey) was contributed by GSH.

It has been reported that the derivatization of thiols with maleimide, including both NEM and NPM (N-(1-pyrenyl) maleimide), will generate diastereomers, some of which can be separated by HPLC, resulting in two peaks [6,21,23,28,29]. Such diastereomer peaks were also observed in this study. Although lengthening the gradient led to better separation of the analytes, it also increased the resolution of the diastereomers for several of the thiol–NEM adducts, which complicated the chromatography. Shortening the gradient led to the co-elution of more analytes (data not shown), which interfered with the selection and monitoring of appropriate mass transitions for each analyte. Therefore, the gradient program was optimized to separate the NEM adducts of the reduced thiols, GSSG, and Met, while allowing diastereomers of a given adduct to co-elute. Under these conditions, only the diastereomeric NEM adducts of Cys-Gly were separable. Although some of the analytes still co-eluted, these analytes could be further differentiated by unique mass transitions (Table 1 and Figure 2). A crosstalk was observed as the GSH fragment ion (due to in-source fragmentation) was isobaric with the Cys-Gly parent ion (*m/z* 304). However, the baseline separation of GSH (retention time 5.41 min) and Cys-Gly allowed the unambiguous assignment of the Cys-Gly twin peaks at retention times of 5.11 and 5.18 min. 

After optimization of chromatographic and MS/MS parameters, the linearity and limit of quantification (LOQ, S/N > 10) were determined for each of the analytes (Table 1). The linear range for each analyte extended over at least three orders of magnitude, except for GSSG. All regression coefficients (R^2^) were greater than 0.99. The limit of quantification differed for each analyte and ranged from 0.01 ng/mL for GSH to 5 ng/mL for GSSG, indicating that the sensitivity of this method is comparable or superior to that of other methods for the analytes investigated here [20,21,30,31,32,33]. 

Spike recovery tests were performed at two levels for each analyte by adding known quantities of analyte standard solutions to B3 cell homogenates, prepared in triplicate. Samples were processed, and analyte concentrations were determined as described above. As shown in Table 2, recoveries ranged from 88% to 126% for both spike levels. RSD for all analytes was ≤12%, indicating that the developed method yields consistent, repeatable results in real-life sample matrices.

### 3.2. Comprehensive Thiol Profiling of Different Cell Lines

**Figure 3:**
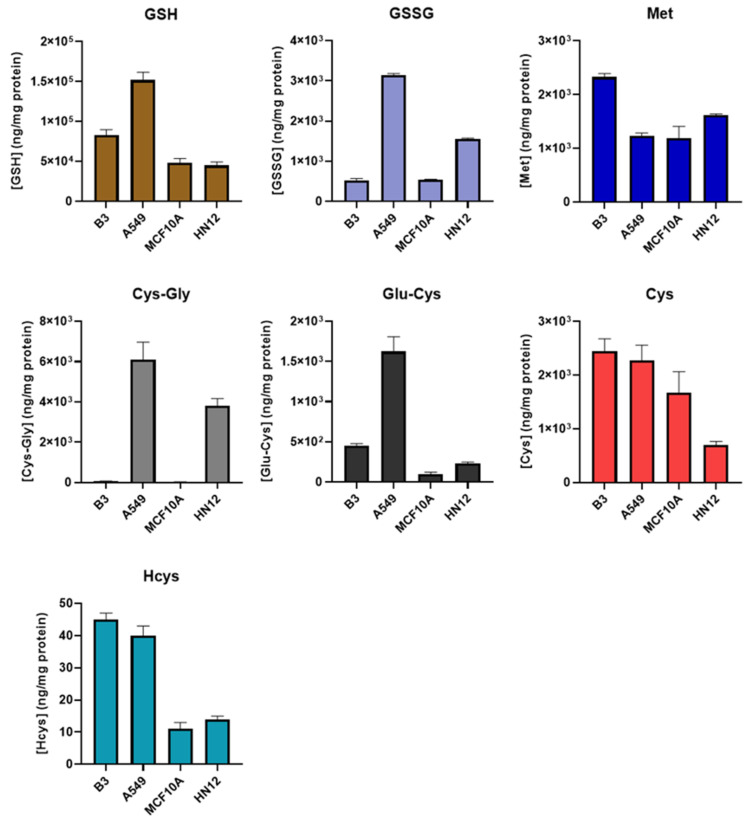
Comparison of thiol profile in different cells. B3, human lens epithelial cells, *n* = 6; A549, human lung carcinoma cells, *n* = 6; MCF10A, human breast epithelial cells, *n* = 3; HN12, human head and neck squamous cell carcinoma line, *n* = 3. Values are reported as mean ± SD.

The newly developed method was then applied to obtain the thiol profiles of four cell lines derived from different tissues. As shown in Figure 3 and Table S1, the resulting profiles from each cell line differed substantially from each other. We observed that the GSH level of A549 cells in this study was similar to that obtained in our previous study [26], in which NEM derivatization was not performed, indicating that the derivatization step likely has little or no effect on the protein quantification. Principal component analysis revealed distinct clusters of profiles obtained from the same cell line (Figure S1). This suggests that the thiol profile may be a distinguishing characteristic of a particular cell line or tissue and may be useful as a metabolic “fingerprint”, and deviations may be indicative of oxidative stress, a property that could be exploited to develop new diagnostics. For example, the thiol profile could be used to isolate and/or identify circulating tumor cells for minimally invasive “liquid biopsies”.

Among all the analytes, Met levels were observed to be the most consistent across the different cell lines, while the greatest variance was observed in Cys-Gly levels. The levels of Cys-Gly in the breast (MCF10A) and lens (B3) epithelial cell lines were approximately 100 times lower than those in head and neck (HN12) and lung (A549) epithelial lines. This is not likely attributable to the degree of tumorigenicity, as the low Cys-Gly levels were observed in other MCF10 lines with 25% and 100% tumorigenicity (data not shown). Energy metabolism and ATP production, in particular, are significantly disrupted in most cancers, and how this impacts the thiol profile may warrant further study [34]. It is also worth noting that the results obtained in this study were from cells grown in a standard incubation chamber. The oxygen level in vivo may be different, leading to possible deviations from the thiol profiles reported here.

### 3.3. Assessment of Intracellular Redox Status in A549 Cells with tBHP Treatment

**Figure 4:**
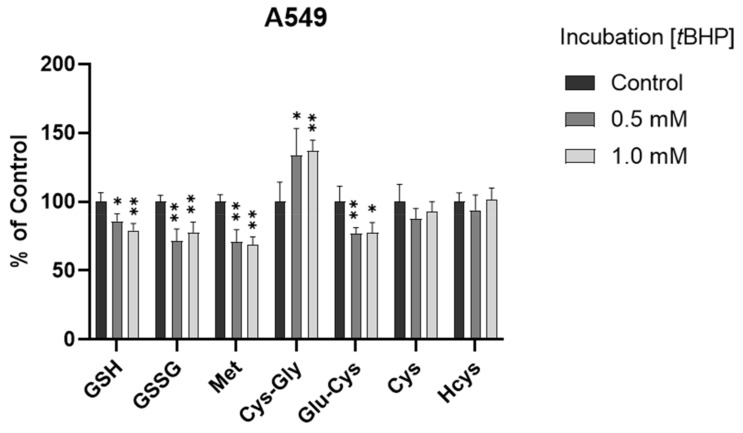
Alteration of analytes in A549 cells after 2 h *t*BHP treatment (*n* = 6). The levels in treatment groups were normalized to those of the control group. *, *p* < 0.05; **, *p* < 0.001.

To determine the ability of the method to monitor changes in thiol profile resulting from oxidative insult, we exposed A549 cells to *t*BHP, a lipid peroxide prototype used to induce oxidative stress in vitro. Different concentrations (0.5 mM and 1 mM) of *t*BHP were applied. After a 2 h treatment, the GSH levels were decreased significantly in both treatment groups (*p* < 0.05 and *p* < 0.001, Figure 4 and Figure S2), consistent with our previous results obtained using HPLC with fluorescence detection [26]. The decrease in GSSG levels may be attributed to the conversion of GSSG to GSH with the upregulation of the GSH recycling pathway under oxidative stress [4,35]. Moreover, Met levels were significantly decreased (*p* < 0.001), Cys-Gly levels were significantly increased (*p* < 0.05 or 0.001), and Cys levels were generally unchanged (*p* > 0.05) in the treatment groups. It was deduced that the unchanged Cys levels could be attributed (or partially attributed) to the conversion of Met to Cys via the transsulfuration pathway and the conversion of GSH to Cys via the GSH degradation pathway. The different changes in GSH and Cys indicated that, compared to the conversion of Cys to GSH via the GSH synthesis pathway, maintaining the Cys level may be more efficient and/or prioritized in the experimental conditions of oxidative stress in A549 cells.

### 3.4. Assessment of Intracellular Redox Status in B3 Cells with tBHP Treatment

In a manner similar to the treatment of A549 cells with *t*BHP, we also exposed B3 cells to *t*BHP at 0.5 and 1.0 mM for 2 h. However, the depletion of GSH was not observed after 2 h in the B3 cells. This unusual observation drove us to perform kinetic thiol profiling in B3 cells with 0.5 mM *t*BHP treatment at different time points (1, 2, and 4 h).

**Figure 5:**
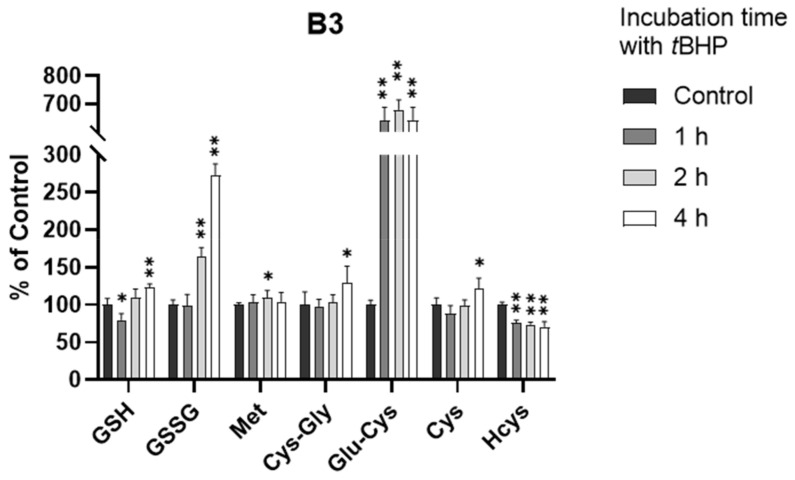
Alteration of analytes in B3 cells after 0.5 mM *t*BHP treatment (*n* = 6). The concentrations in treatment groups were normalized to that of control group. *, *p* < 0.05; **, *p* < 0.001.

As shown in Figure 5 and Figure S3, the GSH level did decrease within 1 h (*p* < 0.05), consistent with our understanding of *t*BHP-induced GSH depletion. However, the GSH level quickly rebounded back to the normal level within 2 h and showed a significant increase at 4 h (*p* < 0.001), indicating that B3 cells may have an efficient defense mechanism against oxidative stress. By investigating the kinetic alterations of all analytes, we believe that this defense mechanism was highly associated with the production of the GSH precursor, Glu-Cys. As shown in Figure 5, the level of Glu-Cys increased more than five-fold after the exposure of B3 cells to oxidative stress. This increase occurred within 1 h of *t*BHP treatment and lasted for over 4 h, supporting a high GSH level after the exposure of B3 cells to oxidative stress. This is surprising since it is well-known that the synthesis of Glu-Cys from Cys catalyzed by glutamate cysteine ligase (GCL) is the rate-limiting step of GSH synthesis [30]. A few studies showed that GCL activity was regulated at the transcriptional and post-transcriptional level in response to oxidative stress [36,37,38,39,40], which might be the reason for the increased Glu-Cys level in this study. However, none of these studies directly observed the change at the metabolite level. Therefore, the connection between the increased level of Glu-Cys and the upregulation of GCL activity in response to oxidative stress in B3 cells remains uncertain and needs to be further investigated. Nevertheless, to the best of our knowledge, this is the first presentation of an oxidative stress defense mechanism in B3 cells via the synthesis of excess Glu-Cys. 

## 4. Conclusions

In this study, a highly sensitive and high-throughput HPLC-MS/MS method was developed to simultaneously determine seven sulfur-containing metabolites that are related to redox status in cultured cells. Subsequent applications showed that the thiol profiles in various cell lines were substantially different from each other. The distinctive features of thiol profiles may be used to generate a “fingerprint” for a given tissue, against which patient samples could be compared for diagnostic purposes. Moreover, the application of this method to *t*BHP-treated A549 and B3 cells revealed different metabolic responses to the oxidative stress. Of particular interest is the quick recovery of GSH levels and high Glu-Cys levels in the human lens epithelial cells; this observation may reveal a robust antioxidant defense mechanism that may have utility in the prevention or treatment of oxidative-stress-related ocular diseases such as cataract.

## Figures and Tables

**Table 1:** HPLC-MS/MS method parameters and performance.

Analyte	Ion Pairs	DP *	CE *	CXP *	LOQ *	Linear Range	R^2^
	(m/z)	(V)	(V)	(V)	(ng/mL)	(ng/mL)	
GSH	433.2 → 304.1	46	20	20	0.01	0.01–500	0.9924
GSSG	613.2 → 355.2	116	29	24	5	5–500	0.9945
Cys-Gly	304.1 → 201.0	66	21	12	0.1	0.1–500	0.9992
Glu-Cys	376.1 → 247.1	71	21	12	0.05	0.05–500	0.9979
Met	150.2 → 103.8	41	15	8	0.1	0.1–500	0.9973
Hcys	261.1 → 55.8	51	41	8	0.05	0.05–500	0.9956
Cys	247.1 → 229.8	51	19	14	0.05	0.05–500	0.9926

* DP, declustering potential; CE, collision energy; CXP, collision cell exit potential; LOQ, limit of quantification (S/N >10).

**Table 2:** Spike recovery and relative standard deviation (RSD, *n* = 3).

Analyte	Recovery (%)	RSD (%)	Recovery (%)	RSD (%)
	Spike level of 100 ng/mL		Spike level of 500 ng/mL	
GSH	115	5	103	1
	Spike level of 4 ng/mL		Spike level of 100 ng/mL	
Cys-Gly	97	9	105	4
Glu-Cys	95	3	103	2
Met	88	3	100	4
Cys	92	12	105	2
GSSG	126	7	124	4
Hcys	105	1	113	2

## Data Availability

Data are contained within the article or supplementary material.

## Supplementary Materials

The following are available online at https://www.mdpi.com/article/10.3390/antiox11010024/s1, Figure S1: Principal component analysis of thiol profiling of different cells. Figure S2: Alteration of analytes in A549 cells after 2 h tBHP treatment (*n* = 6). Figure S3: Alteration of analytes in B3 cells after 0.5 mM tBHP treatment (*n* = 6). Table S1: Concentrations of analytes in different cell lines (mean ± SD, ng/mg protein, *n* = 3).
