# Supplementary material for: Extensive Thiol Profiling for Assessment of Intracellular Redox Status in Cultured Cells by HPLC-MS/MS
Source: Antioxidants (Basel). 2021 Dec 23;11(1):24. doi: 10.3390/antiox11010024 (PMC8773739; doi:10.3390/antiox11010024)
Supplement: Supplementary file 1 [file antioxidants-11-00024-s001.zip › antioxidants-1509362-supplementary.pdf]

Extensive thiol profiling for assessment of intracellular redox status in cultured cells by HPLC-MS/MS

Jiandong Wu[1], Anna Chernatynskaya[1], Annalise Pfaff[2], Huari Kou[1], Nan Cen[3], Nuran Ercal[2*], and Honglan Shi[2*]

[1] Department of Chemical and Biochemical Engineering, Missouri University of Science and Technology, Rolla, Missouri 65409, United States

[2] Department of Chemistry, Missouri University of Science and Technology, Rolla, Missouri 65409, United States

[3] Department of Computer Science, Missouri University of Science and Technology, Rolla, Missouri 65409, United States

**Corresponding authors*

Nuran Ercal
Department of Chemistry
Missouri University of Science and Technology
400 W 11th Street
Rolla, MO 65409, USA
E-mail: nercal@mst.edu
Tel: 573-341-6950

Honglan Shi
Department of Chemistry
Missouri University of Science and Technology
400 West 11th Street
Rolla, MO 65409, USA
E-mail: honglan@mst.edu
Tel: 573-341-4433

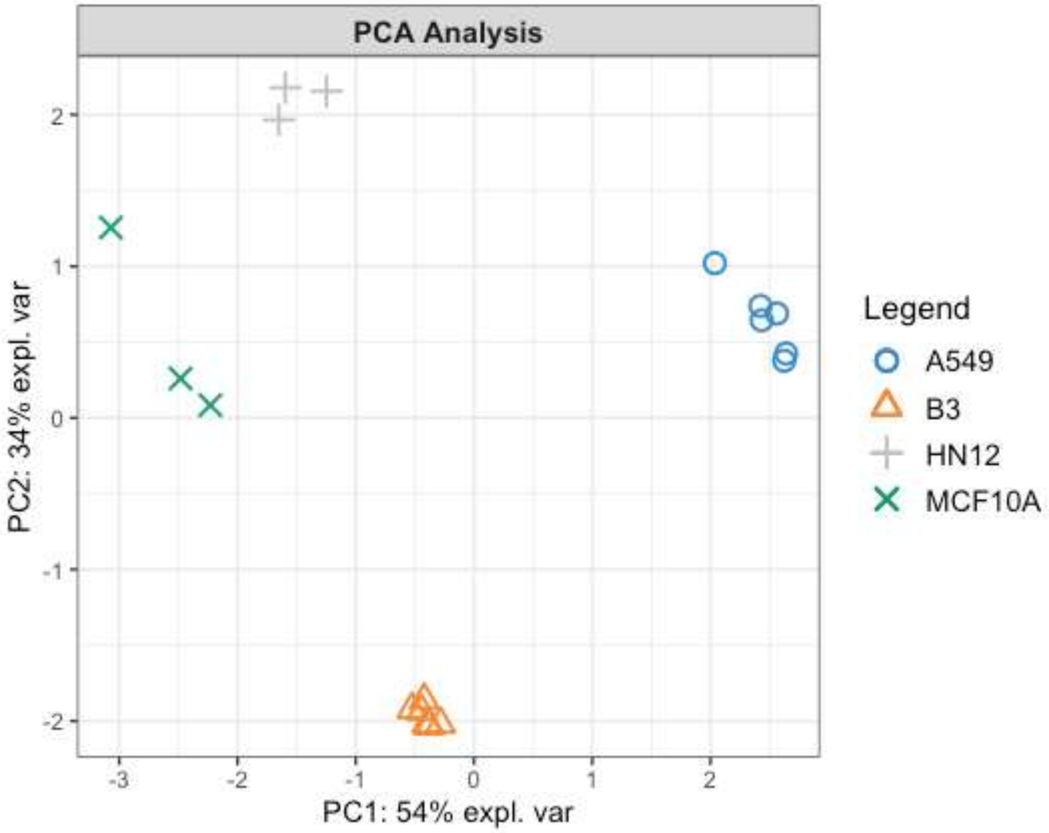

Figure S1. Principal component analysis of thiol profiling of different cells.

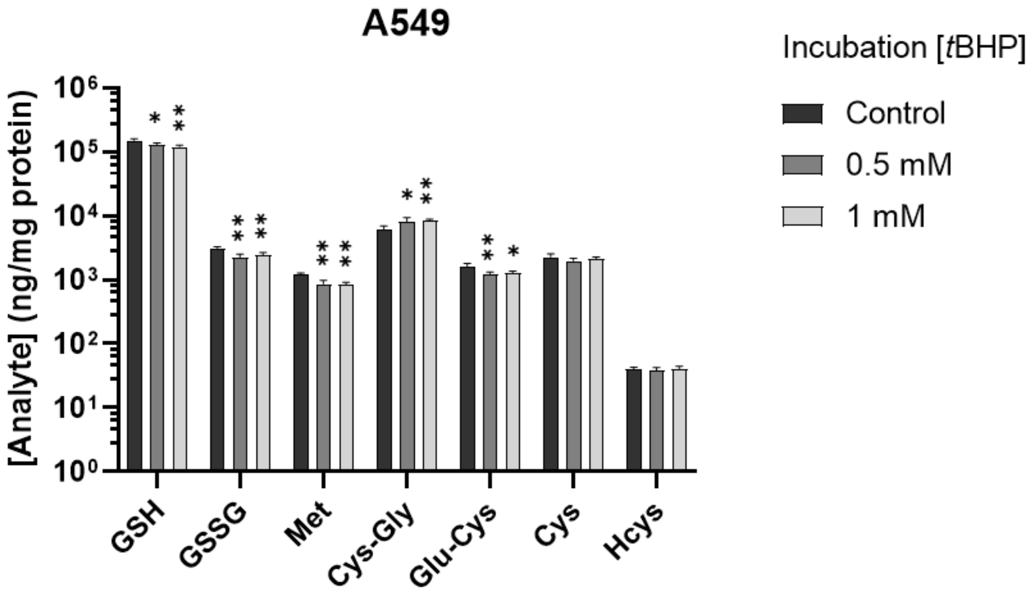

Figure S2. Alteration of analytes in A549 cells after 2-hour *t*BHP treatment (n=6). *, $p < 0.05$; **, $p < 0.001$.

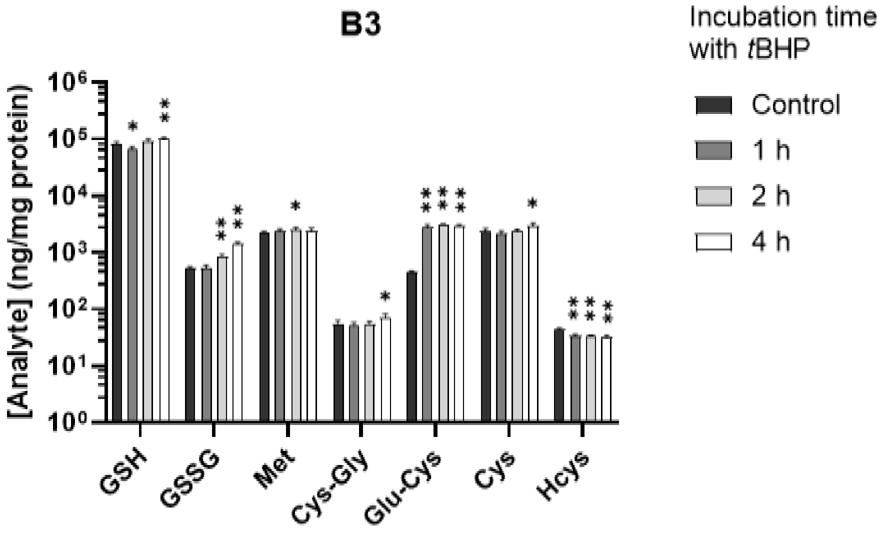

Figure S3. Alteration of analytes in B3 cells after 0.5mM *t*BHP treatment (n=6). *, $p < 0.05$; **, $p < 0.001$.

Table S1. Concentrations of analytes in different cell lines (mean ± SD, ng/mg protein, n = 3).

Tissue	Lens	Lung	Breast	Head/Neck
Cell line	B3*	A549*	MCF10A	HN12
GSH**	83±7	152±10	48±5	45±5
GSSG	527±34	3145±150	543±41	1561±262
Cys-Gly	54±9	6100±868	25±5	3800±364
Glu-Cys	450±28	1624±183	99±21	233±14
Met	2326±66	1225±66	1191±217	1618±22
Cys	2447±229	2275±285	1678±388	697±66
Hcys	45±2	40±3	11±2	14±1

*, n = 6 for B3 and A549; **, μg/mg protein.
